# Machine learning models for prediction of co-occurrence of diabetes and cardiovascular diseases: a retrospective cohort study

**DOI:** 10.1007/s40200-021-00968-z

**Published:** 2022-01-12

**Authors:** Ahmad Shaker Abdalrada, Jemal Abawajy, Tahsien Al-Quraishi, Sheikh Mohammed Shariful Islam

**Affiliations:** 1 Faculty of Computer Science and Information Technology, Wasit University, Al Kut, Iraq; 2 School of Information Technology, Deakin University, Melbourne, Victoria Australia; 3 Institute for Physical Activity and Nutrition, Deakin University, 221 Burwood Highway, Burwood, Melbourne, VIC 3125 Australia

**Keywords:** Diabetes mellitus, Cardiovascular disease, Multi-diseases prediction, Classification and regression, Co-morbidity

## Abstract

**Background:**

Diabetic mellitus (DM) and cardiovascular diseases (CVD) cause significant healthcare burden globally and often co-exists. Current approaches often fail to identify many people with co-occurrence of DM and CVD, leading to delay in healthcare seeking, increased complications and morbidity. In this paper, we aimed to develop and evaluate a two-stage machine learning (ML) model to predict the co-occurrence of DM and CVD.

**Methods:**

We used the diabetes complications screening research initiative (DiScRi) dataset containing >200 variables from >2000 participants. In the first stage, we used two ML models (logistic regression and Evimp functions) implemented in multivariate adaptive regression splines model to infer the significant common risk factors for DM and CVD and applied the correlation matrix to reduce redundancy. In the second stage, we used classification and regression algorithm to develop our model. We evaluated the prediction models using prediction accuracy, sensitivity and specificity as performance metrics.

**Results:**

Common risk factors for DM and CVD co-occurrence was family history of the diseases, gender, deep breathing heart rate change, lying to standing blood pressure change, HbA1c, HDL and TC\HDL ratio. The predictive model showed that the participants with HbA1c >6.45 and TC\HDL ratio > 5.5 were at risk of developing both diseases (97.9% probability). In contrast, participants with HbA1c >6.45 and TC\HDL ratio ≤ 5.5 were more likely to have only DM (84.5% probability) and those with HbA1c ≤5.45 and HDL >1.45 were likely to be healthy (82.4%. probability). Further, participants with HbA1c ≤5.45 and HDL <1.45 were at risk of only CVD (100% probability). The predictive accuracy of the ML model to detect co-occurrence of DM and CVD is 94.09%, sensitivity 93.5%, and specificity 95.8%.

**Conclusions:**

Our ML model can significantly predict with high accuracy the co-occurrence of DM and CVD in people attending a screening program. This might help in early detection of patients with DM and CVD who could benefit from preventive treatment and reduce future healthcare burden.

## Introduction

The prevalence and burden of chronic diseases including diabetes mellitus (DM), cardiovascular diseases (CVD), chronic respiratory diseases and cancers have been increasing over the past three decades in many countries worldwide [1, 2]. Globally, there are 415 million individuals with DM (8.8% of the total world’s population) and the International Diabetes Federation predicts that the number of people with DM will increase to 642 million by 2040 [3]. Similarly, CVD is the leading cause of disease burden in the world [4] and attributes to 17.7 million deaths annually [5]. The prevalence of CVD nearly doubled from 271 million in 1990 to 523 million in 2019 [4]. During the same period the number of CVD deaths also increased from 12.1 million to 18.6 million [4].

Comorbidity is a common problem in many people with chronic diseases such as individuals with DM commonly present with obesity, hypertension, dyslipidaemia and CVD. There are ample evidences of the association between DM and CVD [6, 7]. Both CVD and DM share similar cardiometabolic, behavioral, environmental, and social risk factors. For example, most of the CVD risk factors such as hypertension, obesity, and dyslipidaemia are common in people with DM [7–9]. In contrast, DM is a primary risk factor for CVD [10]. The abnormalities in physiological factors of CVD or DM often result in more than one disease at the same time [11]. People with diabetes have shown to have poor quality of life, increased healthcare expenditure and suffer from more depressive symptoms compared to those without diabetes [12–15]. The comorbid presence of DM and CVD significantly contributes to the increased complications and death [16, 17]. People with DM and CVD are 1.7 times more likely to die compared to those suffering from CVD only [18]. Moreover, both CVD and DM are directly associated with cardiovascular autonomic neuropathy which can increase complications and deaths [19, 20].

DM and CVD often remains undetected in the early phases of the disease and therefore untreated, leading to complications and premature deaths. Therefore, it is essential to provide a practical and viable model to predict these diseases early together in order to reduce the future morbidities and premature deaths. A number of studies have used ML approaches to predict DM and CVD in different populations and using different methods [21–25]. However, evidence on ML approaches for predicting co-occurrence of DM and CVD is lacking. Perceiving the common risk factors and developing a predictive model for co-occurrence of DM and CVD is more important for prevention and management of these diseases than targeting individual diseases. Therefore, in this study we aimed to identify the common risk factors for DM and CVD, develop a multi-diseases predictive model capable of predicting DM and CVD simultaneously and evaluate the performance of the prediction model.

## Materials and methods

### Design

We conducted secondary analysis from a retrospective cohort study. We developed a two-stage approach to predict the occurrence of DM and CVD comorbidities based on their common risk factors. In the first stage, logistic regression (LR) and the Evimp functions (EVF) were implemented in Multivariate Adaptive Regression Splines (MARS) model to infer the significant common risk factors based on voting criteria. Afterward, the correlation matrix is applied to reduce the redundancy of common risk factors. In the second stage, Classification and Regression (CART) algorithm is employed in constructing a predictive model of DM and CVD.

### Participants, location and data collection

We used the diabetes complications screening research initiative (DiScRi) datasets which contains data from 2000 participants on more than 200 variables collected from Charles Stuart University in New South Wales, Australia from 2004 [26]. Patients were recruited through a public media campaign, including newspaper, radio, local television, and advertisements posted in general practice and community health centres. People were requested to contact the university if they wished to undergo a health check, and an appointment was made to attend the clinic. All participations older than 40 years were eligible to participate [27]. Participants with existing cardiovascular, respiratory and renal disease as well as depression, schizophrenia and Parkinson’s disease were excluded. The data collection procedure involved the following steps: (1) all participants were required to stop smoking or to consume drinks like alcohol and coffee 24 h before being tested. They were required to fast, beginning from midnight prior to the testing day. The tests were conducted from 9:00 am to 12:00 pm.

### Ethics

Written informed consent was obtained from all participants before data collection. The protocol for the DiScRi study was approved by the Ethics in Human Research Committee of the Charles Sturt University (Protocol # 03/164).

### Variables and measurements

The DiScRi dataset contain data on participants sociodemographic information, diseases history, measurements of blood pressure (BP), heart rate, electrocardiograms, blood biochemistry tests and Ewing battery tests. In this study we used the following variables: family history (FH) of disease, gender, age, body mass index (BMI) measured as height in cm divided by weight in kg^2^ waist circumference, hypertension status (yes or no), glycated hemoglobin (HbA1c), blood lipid profile including triglyceride (TG), Total cholesterol (TC), High-density lipoproteins (HDL), Low-density lipoproteins (LDL) and ratio of total cholesterol to high-density lipoprotein (TC/HDL ratio). Blood glucose estimations included fasting glucose test (FGT) and Glucose Tolerance Test (GTT) measured in (mmol/L).

Finally, we recorded the DM and CVD status of the participants and conducted Ewing’s Test including: 1. Lying to standing heart rate (LSHR) change expressed by 30:15 ratio. Such test indicates to the ratio of longest R-R interval (ranged from 20 to 40 beat) to the shortest R-R interval (ranged from 5 to 25 beat) produced by a change in position (from a horizontal position to vertical position); 2. Deep breathing heart rate (DBHR) change, which refers to the evaluation of beat-to-beat Heart Rate variation (R-R variation) based on deep breathing; 3. Valsalva maneuver heart rate (VAHR) change measuring the response of heart rate during and after increasing the intra-abdominal and intrathoracic pressure; 4. Handgrip blood pressure (HGBP) change measuring the change in diastolic BP after using a handgrip dynamometer; and 5. Lying to standing blood pressure (LSBP) change measuring the difference in the baroreflex-mediated BP after a change in the position.

### ML models: DM and CVD comorbidity predictive model

**Fig. 1:**
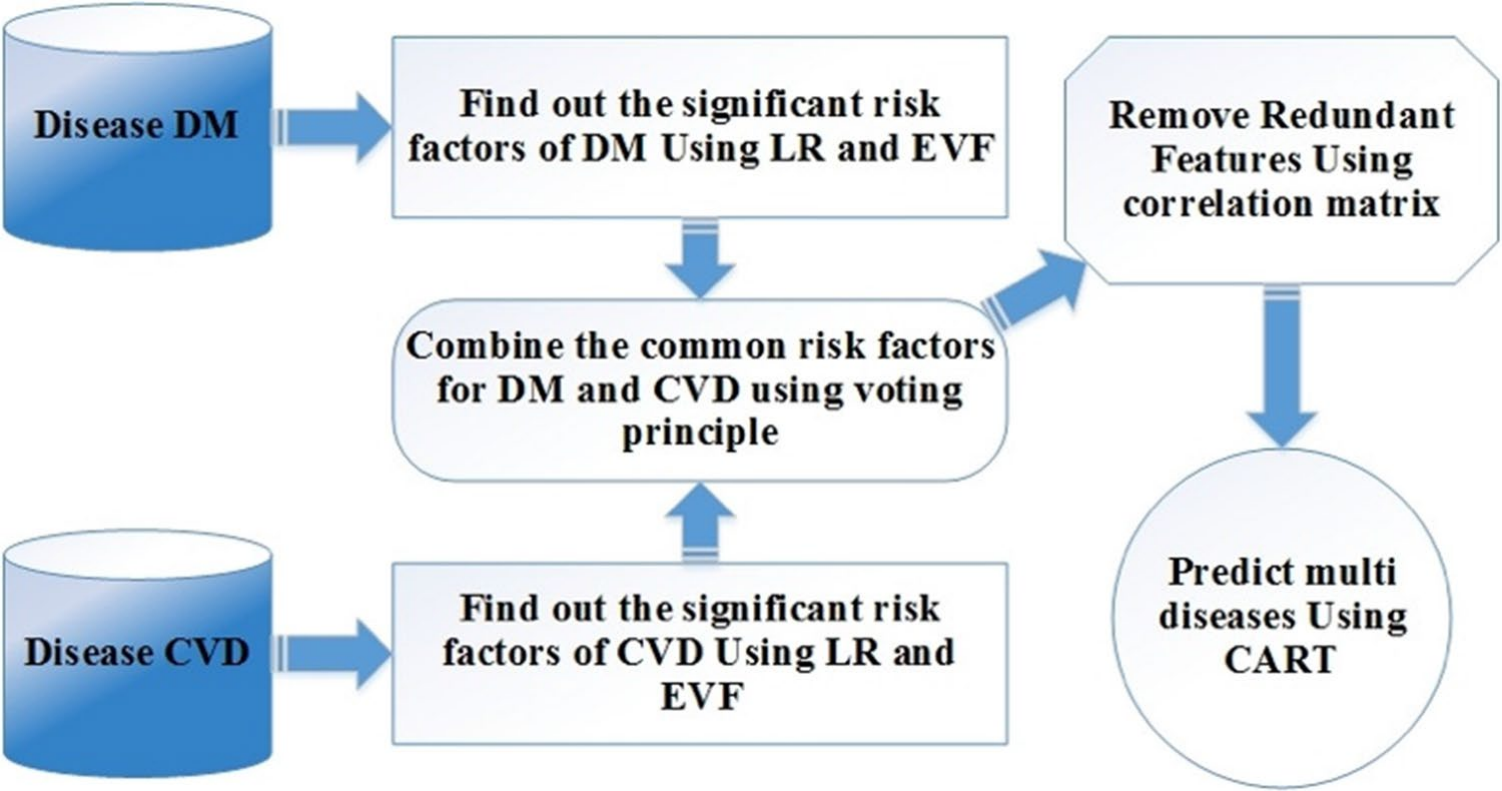
The Framework of the Predictive Model

Figure 1 illustrates the proposed model, which consists of two main stages. The first stage focuses on the extraction of the common risk factors from the dataset. The output of the first stage becomes the input of the second stage, which deals with predicting the co-occurrence of the two diseases. In the following subsections, the two stages are explained in detail.

### Data analysis

#### Extracting common risk factors

The common risk factors refer to all factors that show a significant association in both diseases. We used two feature selection methods to extract the common risk factors of DM and CVD: logistic regression (LR) [28], and the Evimp function (EVF) implemented in multivariate adaptive regression splines model (MARS) [29]. These methods were chosen due to their efficiency in determining the association between the independent variables and the outcome [30–32].

### Logistic regression

Logistic Regression (LR) model computes the probability of occurrence of dependent variable based on the predictability of independent variables. In general, the LR can be expressed as follows:

1 $$\log \left(\frac{\pi }{1-\pi}\right)={\beta}_0+{\beta}_1{x}_1+{\beta}_2{x}_2+\dots {\beta}_n{x}_n$$

where *π* represents the probability of occurrence of an outcome (dependent variable) based on the selected independent variables. *β* indicates the regression coefficients of each independent variables. In this paper, we use logistic regression with forward stepwise method to select the significant risk factors with (P < 0.05).

### Estimate variable importance implemented in MARS

**The Evimp function (EVF)** is a method implemented in multivariate adaptive regression splines model (MARS) [29]. The EVF returns a matrix presenting the relative significance of the features in the model and uses three criteria in estimating the importance of features as follows:

- The (nsub-set) criterion calculates the number of sub-sets that involves the feature. Features that are involved in more sub-sets are considered most important.
- The raw residual sum-of-squares (RSS) criterion contains two steps. Firstly, it computes the reduction in the RSS for each sub-set and compares the reduction with the value of the previous subset. After, for each involved feature, RSS aggregates these reductions for all sub-sets that involve the feature. In the end, the total aggregation of reductions is interpreted. Features that cause a massive reduction in RSS are most important.
- The generalised cross-validation (GCV) criterion is similar to RSS criterion; however, it uses GCV instead of RSS. GCV evaluates the performance features in sub-sets and selects the most significant sub-set (lower values of GCV are useful).

#### Voting method

After identifying the risk factors (statistically significant) for each disease by LR and EVF, a voting method was applied to determine the common risk factors of DM and CVD. The correlation matrix method was then performed to remove the redundant common risk factors and avoid the problem of multicollinearity using cut-off = 0.5.

### Comorbidity predictive model

In the second stage, classification and regression (CART) algorithm [33] was used to construct the predictive model of DM and CVD comorbidity based on the extracted common risk factors from the first stage. CART was used due to its several advantages. For example, in comparison with other ML algorithms, CART outcomes are easy to interpret visually using If-then condition, which is like a human decision. It does not require specifying the association between independent variables and the outcome. CART can handle both classification and regression problems. Furthermore, CART is capable of dealing with the continuous and discrete dependent variables. It automatically amends the constructed tree to reduce the impacts of the measured impurities and identifies the efficiency of the node for a final decision. In general, CART algorithm uses Gini index to build the decision trees. Gini index measures the impurity or purity of the features. The general formula Gini index used by CART is as follows:

2 $$Gini\ index=1-\sum_{i=1}^{\mathrm{j}}\ {p}_i^2$$

In fact, the initially constructed tree in Eq. (2) does not represent the optimal result. Therefore, CART algorithm prunes the created tree using node error rate as follows:

3 $${node}_{error\ rate}=X{e}_i/\sum_{i=1}{X}_i$$

where *Xe*_*i*_ gives the number of misclassified instances in the node, and $\sum_{i=1}{X}_i$ provides the total number of instances in the node. The process of pruning tree starts from bottom to top based on the total error rate condition. If the rate of total error is higher than the stated threshold, then it stops the process of pruning.

#### Evaluation of the predictive model

We evaluated the multi-stage comorbidity prediction model using various measures such as accuracy, sensitivity, specificity and confusion matrix measurements. The evaluation measures used are as follows:

- ***Sensitivity:*** defines the number of participants that are correctly predicted with the positive disease.

4 $$Senstivity=\frac{TP}{FN+ TP}$$

- ***Specificity:*** refers to the number of participants that are correctly predicted with the negative disease.

5 $$Specificity=\frac{TN}{FP+ TN}$$

- ***Accuracy:*** exposes the total number of participants that are correctly predicted with the positive and negative diseases.

6 $$Accurcy=\frac{TP+ TN}{\left( Total\ nubmer\right)}$$

The 10-fold cross-validation (CV) approach was applied to obtain a balanced evaluation of the generalisation error. Cross-validation is an approach used to evaluate the performance of predictive models by partitioning the entire dataset into k number of sub-sets. It uses 10-fold cross-validation to randomly divide the entire dataset into ten sub-sets; 9 sub-sets are used for training stage (90%), and the remaining sub-set is used for the testing stage (10%) with replacement in the sub-sets. The hardware used are: Intel Core i9 10850K 3.6Ghz Comet Lake 10 Core 20 Thread LGA1200, GeForce RTX 3070 GAMING Z TRIO 8G LHR GRAPHIC CARD and MPG Z590 Gaming Edge WiFi LGA1200 ATX Desktop Motherboard. Data were analyzed using SPSS version 20.0 (SPSS, Inc., Chicago, IL, USA) and R language.

## Results

A total of 812 participants were included in this study (244 with CVD, 237 with DM, 139 with CVD and DM simultaneously, and 192 healthy disease-free participants).

### Common risk factors of DM and CVD

**Fig. 2:**
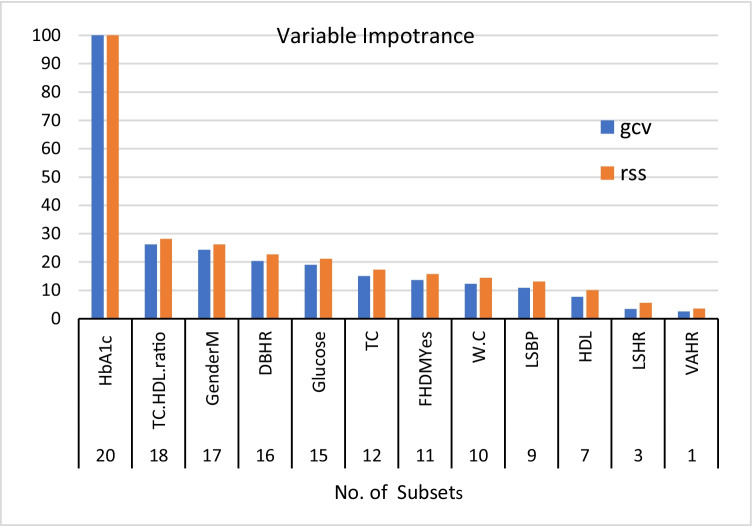
Essential Features of DM by EVF. Note: HbA1c glycated hemoglobin, TC Total cholesterol, HDL High-density lipoproteins, LDL Low-density lipoproteins, DBHR Deep breathing heart rate, FHDM Family History of DM, W.C Waist Circumference, LSBP Lying to standing blood pressure, VAHR Valsalva maneuver heart rate

Table 1 shows the risk factors of DM selected by LR and the Evimp function (EVF). In the LR model family history of diabetes, gender, age, lying to standing heart rate change, deep breathing heart rate change, lying to standing BP change, HbA1c, FGT, TC, HDL and TC\HDL ratio were significantly associated with DM (P < 0.05). The outcomes of applying an Evimp function (EVF) with DM demonstrates that family history of diabetes, gender, waist circumference, lying to standing heart rate change, deep breathing heart rate change, Valsalva maneuver heart rate change, lying to standing BP change, HbA1c, FGT, TC, HDL and TC\HDL ratio were the most important features. Figure 2 illustrates the features’ importance of DM by Evimp function (EVF). Risk factors that were common in both LR and EVM models are shown in Table 1.

**Table 1:** Summarizes DM risk factors under LR and EVF

Covariates	LR	(EVF)	Risk factors of DM
FH	*	*	*
Gender	*	*	*
Age	*		
W.C		*	
BMI			
LSHR	*	*	*
DBHR	*	*	*
VAHR		*	
HGBP			
LSBP	*	*	*
HbA1c	*	*	*
Triglyceride			
Glucose	*	*	*
TG			
HT Status			
TC	*	*	*
HDL	*	*	*
TC\HDL ratio	*	*	*
LDL			

*LR* Logistic Regression, *EVF* Evimp functions, *FH* Family History, *W.C* Waist Circumference, *BMI* Body Mass Index measured in kg/m^2^, *LSHR* Lying to standing heart rate, *DBHR* Deep breathing heart rate, *VAHR* Valsalva maneuver heart rate, *HGBP* Handgrip blood pressure, *LSBP* Lying to standing blood pressure, *HbA1c* glycated hemoglobin, *TG* triglyceride, *HT* Hypertension, *TC* Total cholesterol, *HDL* High-density lipoproteins, *LDL* Low-density lipoproteins

**Fig. 3:**
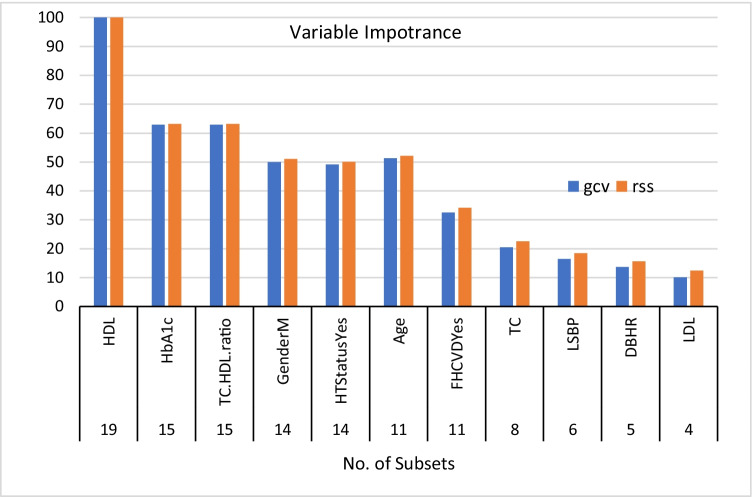
Essential Features of CVD by EVF. Note: HDL High-density lipoproteins, HbA1c glycated hemoglobin, TC Total cholesterol, HDL High-density lipoproteins, HT Hypertension, FHCVD Family History of CVD, LSBP Lying to standing blood pressure, DBHR Deep breathing heart rate, LDL Low-density lipoproteins

Similarly, the risk factors of CVD as selected by logistic regression (LR) and the Evimp function (EVF) are presented in Table 2. Family history of CVD, gender, age, deep breathing heart rate change, lying to standing BP change, HbA1c, hypertension status, TC, HDL and TC\HDL ratio were significantly associated with CVD (P < 0.05). The EVF result shows that family history of CVD, gender, age, deep breathing heart rate change, lying to standing BP change, HbA1c, hypertension status, TC, HDL, LDL and TC\HDL ratio were significantly correlated with CVD. Figure 3 clarifies the feature significance of CVD by using EVF. As shown in Table 3, the common risk factors for both diseases are family history of the disease, gender, deep breathing heart rate change, lying to standing BP change, HbA1c, TC, HDL and TC\HDL ratio. Later, the remove redundant features method was applied and showed there was inversely related correlation between TC feature and TC\HDL ratio with *r* =  − 0.8. Therefore, the TC feature is removed from the common risk factor set. Thus, the final set of common risk factors was FH of the disease, gender, DBHR, LSBP, HbA1c, HDL and TC\HDL ratio.

**Table 2:** Summary of CVD risk factors by LR and EVF

Covariates	LR	(EVF)	Risk factors of CVD
FH	*	*	*
Gender	*	*	*
Age	*	*	*
W.C			
BMI			
LSHR			
DBHR	*	*	*
VAHR			
HGBP			
LSBP	*	*	*
HbA1c	*	*	*
Triglyceride			
Glucose			
TG			
HT Status	*	*	*
TC	*	*	*
HDL	*	*	*
TC\HDL ratio	*	*	*
LDL		*	

*LR* Logistic Regression, *EVF* Evimp functions, *FH* Family History, *W.C* Waist Circumference, *BMI* Body Mass Index measured in kg/m^2^, *LSHR* Lying to standing heart rate, *DBHR* Deep breathing heart rate, *VAHR* Valsalva maneuver heart rate, *HGBP* Handgrip blood pressure, *LSBP* Lying to standing blood pressure, *HbA1c* glycated hemoglobin, *TG* triglyceride, *HT* Hypertension, *TC* Total cholesterol, *HDL* High-density lipoproteins, *LDL* Low-density lipoproteins

**Table 3:** Common risk factors of DM and CVD

Covariates	Risk Factors of DM	Risk Factors of CVD	The common risk factors
FH	*	*	*
gender	*	*	*
Age		*	
W.C			
BMI			
LSHR	*		
DBHR	*	*	*
VAHR			
HGBP			
LSBP	*	*	*
HbA1c	*	*	*
Triglyceride			
Glucose	*		
SG			
HT Status		*	
TC	*	*	*
HDL	*	*	*
TC\HDL ratio	*	*	*
LDL			

*LR* Logistic Regression, *EVF* Evimp functions, *FH* Family History, *W.C* Waist Circumference, *BMI* Body Mass Index measured in kg/m^2^, *LSH*R Lying to standing heart rate, *DBHR* Deep breathing heart rate, *VAHR* Valsalva maneuver heart rate, *HGBP* Handgrip blood pressure, *LSBP* Lying to standing blood pressure, *HbA1c* glycated hemoglobin, *TG* triglyceride, *HT* Hypertension, *TC* Total cholesterol, *HDL* High-density lipoproteins, *LDL* Low-density lipoproteins

### Performance of the proposed model

**Fig. 4:**
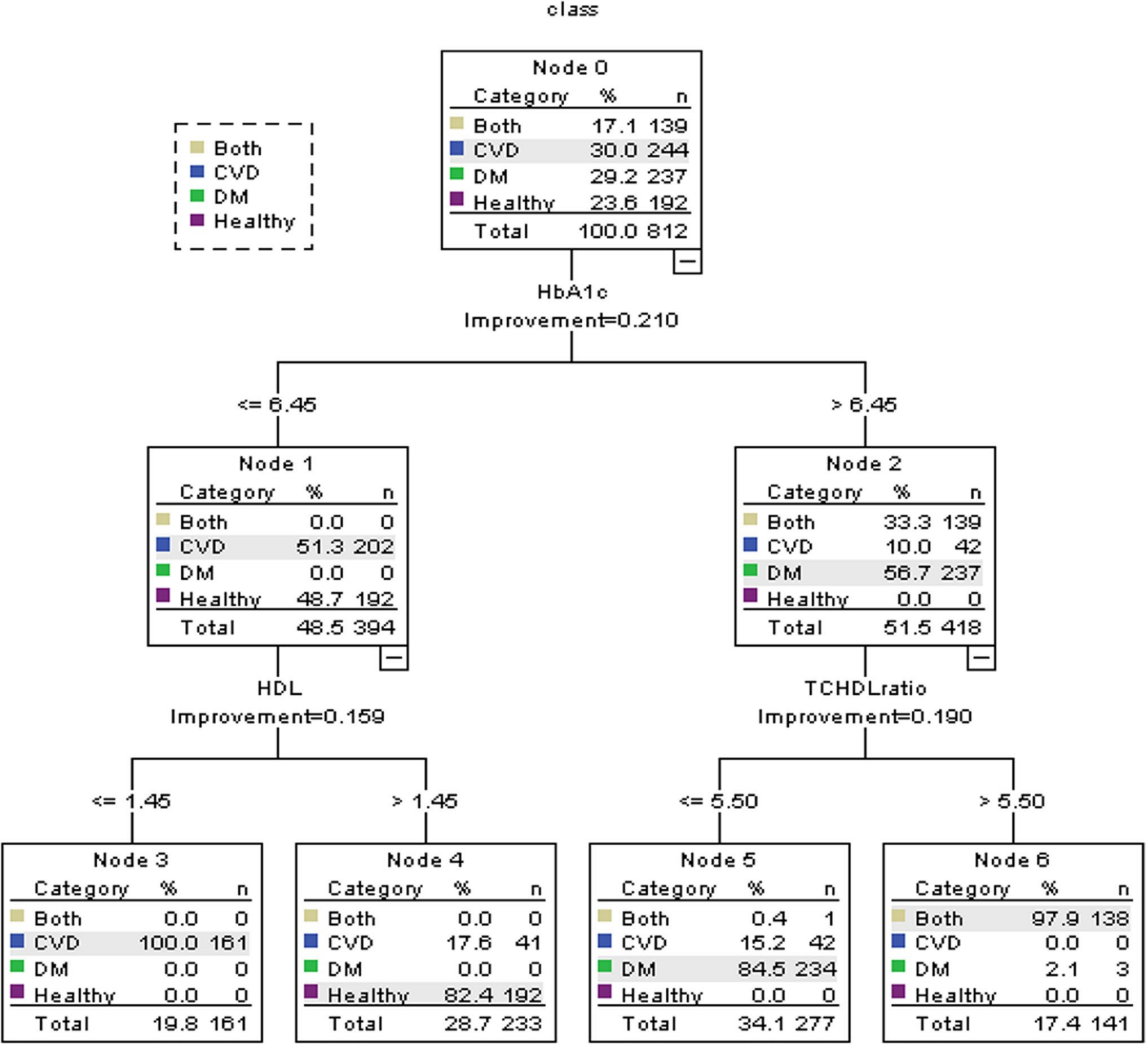
The tree constructed of the proposed model

Figure 4 depicts the obtained tree of the evaluation of participants with the final set of common risk factors of multi-diseases using the predictive model. As shown in Fig. 4, HbA1c, HDL, and TC\HDL ratio risk factors played significant roles in creating the rules of the model. The predictive model showed that the participants with HbA1c >6.45 and TC\HDL ratio > 5.5 are at risk of developing both diseases with probability 97.9%. In contrast, the participants with HbA1c >6.45 and TC\HDL ratio ≤ 5.5 are more likely to gain only DM with probability of 84.5%. Participants with HbA1c ≤5.45 and HDL > 1.45 are likely to fall under the healthy group with probability of 82.4%. The predictive model also showed participants with HbA1c ≤5.45 and HDL <1.45 are at risk of only CVD with probability (100%).

Table 4 summarizes the rules and the diagnosis outcomes of the created tree of multi-diseases. The created rules are straightforward to interpret, and thus physicians can use them to make proper decisions. The multi-diseases prediction outcomes of the validation sample based on the most common risk factors are presented in Table 5 (confusion matrix).

**Table 4:** Induction rules of the proposed model

Node	Rule	Class
6	If HbA1c is >6.45 and TC\HDL ratio > 5.5 Then	Both
5	If HbA1c is >6.45 and TC\HDL ratio ≤ 5.5 Then	DM
4	If HbA1c ≤5.45 and HDL > 1.45 Then	Healthy
3	If HbA1c ≤5.45 and HDL < 1.45 Then	CVD

*HbA1c* glycated hemoglobin, *TC* Total cholesterol, *HDL* High-density lipoproteins

**Table 5:** Confusion matrix of multi-diseases prediction model

Predictive Class				Actual Class
A	B	C	D	
218	12	0	14	A = CVD
9	225	3	0	B=DM
0	2	137	0	C = Both
8	0	0	184	D = Healthy

As evident from the confusion matrix, the performance of the predictive model was very good with an accuracy of 94.09%. Meanwhile, the sensitivity and the specificity of the predictive model were 93.5% and 95.8% respectively. As presented in the confusion matrix, out of 244 there were 26 CVD participants incorrectly predicted. As for DM, the number of incorrectly predicted participants were 12 out of 237. In the same context, out of 192 healthy participants, the model incorrectly predicted eight participants. As for participants with both diseases two out of 139 participants were incorrectly predicted. The outstanding performance of this model is due to all the included tests were significantly associated with both diseases (P < 0.05).

## Discussion

In this study, we developed a two-stage ML model to predict the co-occurrence of DM and CVD based on their common risk factors and evaluated its prediction accuracy, sensitivity and specificity. Our results suggest that a ML model can significantly predict with high accuracy the co-occurrence of DM and CVD in people attending a screening program. Thus, increasing early detection of patients who could benefit from preventive treatment and reduce future healthcare burden. In recent years, several studies have developed predictive models for DM [34–37] and CVD [38–42]. However, the existing models can predict only a single disease (CVD or DM) at a time. Since patients may suffer from multiple related diseases at the same time, these models are inadequate for predicting the co-occurrence of DM and CVD simultaneously.

A number of studies have developed models for predicting several diseases and comorbidities [9, 43–48]. Chun and colleagues [49] have introduced a comorbidity prediction method using filtering technique to predict likely comorbid conditions for individuals and a trajectory prediction graph model to reveal progression paths of the conditions. A recent work [50] used social network patient data as evidence-based knowledge to support decision making in disease progression for comorbidities. The proposed model was based on statistical modelling of the constructed knowledge base. However, the work calculated the similarity between a patient’s record and other patients’ records and derived the risk of a certain medical condition using patients self-reported data. Another research [51] proposed a cascade data mining approach for frequent pattern mining enriched with context information, including a new algorithm MIxCO for maximal frequent patterns mining. The work explicated some population specific comorbidities such as schizophrenia, hyperprolactinemia and Type 2 DM. Other studies have identified potential comorbidities based on mutant genes, enzymes and protein-protein interactions [52–55]. A study [56] proposed a two-phase predictive model to simultaneously predict hypertension and hyperlipidaemia based on their common risk factors.

Both DM and CVD share common risk factors. DM is a complex disease influenced by multiple factors like genetics, lifestyle and environmental conditions [57]. Blood tests for HbA1c and glucose are reliable tests for diagnosing diabetes as recommended by the American Diabetes Association and the World Health Organization [58, 59]. High lipid profile including TC, HDL and TC\HDL ratio are significant predictors of DM [60, 61]. As for Ewing’s tests such as LSHR, DBHR, LSBP are the gold standard tests for cardiac autonomic neuropathy which is directly associated with DM. Further, family history of CVD, gender, age, and hypertension status are common risk factors for CVD and used in the Framingham Risk Score [62, 63]. Dyslipidaemia is also a main risk factors for CVD in people with DM [64, 65]. Increase in HbA1c showed progressively increasing risks of CVD [66]. Ewing tests has shown to an independent prognostic indicator of sudden arrhythmic death risk [67]. Abnormalities in HbA1c was associated with both DM and CVD [59, 68, 69]. Compared to LDL cholesterol, the HDL cholesterol level is a robust risk factor for coronary heart disease and DM [70]. The ratio of total cholesterol to HDL is also a risk factor for cardiovascular events [65, 71, 72]. Presence of family history was found to be significantly correlated with the prevalence of both diseases [73, 74].

We found common risk factors for DM and CVD co-occurrence were family history of the diseases, gender, deep breathing heart rate change, lying to standing BP change, HbA1c, HDL and TC\HDL ratio. Our predictive model showed that the participants with HbA1c >6.45 and TC\HDL ratio > 5.5 were at risk of developing both diseases (97.9% probability). In contrast, participants with HbA1c >6.45 and TC\HDL ratio ≤ 5.5 were more likely to have only DM (84.5% probability) and those with HbA1c ≤5.45 and HDL >1.45 were likely to be healthy (82.4% probability). Further, participants with HbA1c ≤5.45 and HDL <1.45 were at risk of only CVD (100% probability). Our results indicate that Ewing’s tests (DBHR and LSBP) could be used in the prediction of DM and CVD co-occurrence.

A major strength of this study in the use of two robust ML models to detect DM and CVD co-occurrence which can be a medical revolutionary. This study has potential limitations that should be considered for interpreting the results. First, data were collected from a small number of participants attending a screening program in a rural health centre in Australia. Therefore, the model might not produce the same results in another setting. Second, data on other important markers of diabetes and CVD for example, heart rate variability, retinal scans, peripheral nerve function, and various parameters derived from electrocardiogram recordings were not available. Third, we did not record the run time for the machine learning modlels which might be useful for its clinical application. Finally, our ML model has not been tested in a clinical population. Future research involving representative participants with larger sample size in multiple clinics with long-term follow-up are needed. Future machine learning studies should attempt to compare the algorithms developed in similar other datasets for better comparisons. Also, there is a need to explore other important cardiovascular and metabolic markers in the model which could improve the prediction power and accuracy.

The analysis and evaluation of the proposed model shows that it is very efficient and seamless to employ and use. The prediction accuracy of existing computational approach also requires to be improved as well as computationally complex. Evaluation of the model in clinics and training healthcare providers to use the ML models will improve the success of DM and CVD screening. Previous research have shown that digital health approaches could be useful for prevention and management of DM and CVD [75, 76]. Evidence suggest that using simple mobile phone services such as text messaging are effective and cost-effective approaches for controlling DM and CVD [77–80]. Our ML models could easily be employed as a tool for web-based and mobile phone application, thus increasing its reach among people with DM, CVD and healthcare providers.

## Conclusion

Our ML model provides high accuracy, sensitivity and specificity, making it potential for utilization by primary healthcare providers in the clinics. Early detection of both DM and CVD will facilitate the planning of timely intervention and creates greater awareness of the risk of the diseases.
